# On Cholera Morbus

**Published:** 1822-09

**Authors:** John Adam

**Affiliations:** Assistant Surgeon to the General Hospital, Calcutta, &c. &c. &c.


ART. V.—*On Cholera Morbus.* By JOHN ADAM, M.D. H.C.S. Assistant Surgeon to the General Hospital, Calcutta, &c. &c. &c. Communicated by Dr. WEBSTER.*

FROM the month of April, 1820, to the 1st of January, 1821, twenty-one cases, in all, of this fatal disorder were admitted into the left ward of the General Hospital. Of these, two were *threatened* cholera, both of whom recovered. They were marked as such because they wanted the characters of the disorder in its complete form, while at the same time some of the peculiar symptoms were present, showing the tendency to it. In one, there was pain across the epigastrium and hypochondriac regions, increased upon pressure, with vomiting, and cramp occasionally of the lower extremities; the attack having been sudden, as in cholera; but the pulse was rather full, though not strong; and there was no sinking and pallor of the countenance, as in the dreaded epidemic; no purging; and the body had not lost much, if any, of its natural heat. The other subject had purging and was affected with cramp, without vomiting or pain at the epigastrium; the surface was warm, and pulse rather full. They were stout seamen, and had been drinking freely. Bleeding to twenty ounces, followed up by mercurial purges, removed the urgent symptoms in both cases. In the first, the pain of the side continuing, with signs of local determination to the liver and lungs, mercurials were given so as to

* The author writes, in a letter to Dr. Webster, that "he has merely detailed, with scrupulous exactness, what he saw, and is recorded in the diary of cases kept at the hospital; and that the statement is to be considered more in the light of a clinical report, than as a regular history of this very severe and formidable disease."

affect the mouth, and afterwards topical bleeding was used; and in thirteen days the patient was discharged from the hospital. The other was perfectly recovered after the second dose of the purgative, and did not remain more than three days altogether in the hospital. It is highly probable that the disorder in both was caused by hard drinking, and exposure to the direct rays of the sun; at least, no other apparent origin could be assigned by them.

All the cases of cholera admitted into the left ward of the hospital of which I was in charge, had existed for several hours, or even for days. They were consequently received, and became the subjects of treatment, at very different periods or stages of the disorder; and it is not to be wondered at, if all our endeavours to recover them generally proved unsuccessful. In a disease where the delay of a single hour in administering the proper remedies may carry the patient beyond the reach of human skill, we could not expect that, after the lapse of many hours, the most active practice would be attended with a favourable result. Accordingly, I never heard of a case of cholera arriving at the hospital, but I crossed over to visit it with a fearful foreboding; and a single glance at the unfortunate patient too frequently served to confirm this presentiment. Where the surface felt cold, and the pulse could barely be perceived at the wrist, with a sunken countenance and weak voice, the case was considered desperate, and medicine had no more effect than if it had been applied to a body already deprived of life.

In reference to the symptoms of the disease at the period of admission, I may describe it as consisting of three different stages, or grades. Whether these occur in the order of succession in every case, I cannot say; but it seems to me probable, were accurate observations made from the first signs of approaching illness exhibited by the patient, that they would be discovered invariably. At all events, it is not departing from the strictest rules of pathological investigation to state the varieties of the disorder, as observed in different individuals, without attempting to draw any inference from the facts as to the cause of these differences.

Cholera, then, displayed itself in three stages or states: 1st, of oppression and excitement; 2d, of oppression and exhaustion; and 3d, of febrile re-action.

*First, of oppression and excitement.*—So seldom have we an opportunity at the hospital of seeing cholera in the commencement, that even the cases which I have selected as instances of the first stage of this disorder had existed, I find, between eight and ten hours each, previously to admission; and it may therefore be believed, that the variation exhibited in the symptoms had depended upon some peculiarity in the individual, rather than the period of the disease. With that view, they may be styled, more properly, cholera with excitement. There was vomiting and purging of a thin, whitish, watery liquid, with severe cramps of the trunk and extremities; countenance anxious; not sunken, (unless a little around the eyes, marked by a blue circle,) but rather inclining to a flush; and the general expression of the patient indicative of some overpowering load about the chylopoietic viscera and heart; pain, but not severe, at the epigastrium; pulse quick, small, and hard, about 120; skin warm, or a little above the natural temperature; much thirst; tongue furred, in one case white. In common with the other cases of the epidemic, some had vomiting, purging, and cramps; pulse distinct and hard; countenance not fallen; heat not reduced; and the veins of the arm, when opened, pouring forth their contents in a continued stream, and nearly of the natural colour. They were all young men between twenty-five and thirty-five. In two cases, bleeding, generally and topically, formed the plan of treatment, followed up by calomel and opium in very liberal doses. The beneficial effects of the bleeding were particularly manifest in one of these, a stout, raw recruit, fresh from Europe, who was brought into the hospital from the garrison, and had been previously bled by the surgeon there. While several of his comrades were dying around him, in whom the lancet was inadmissible, from the state of the symptoms, this poor fellow received renovated vigour and ease from every additional ounce of blood drawn from his arm, and expressed the great relief he felt during the time the operation was going forward. He took, afterwards, half a drachm of calomel and ten grains of opium, with a drachm of tincture of lavender; and had an enema, with half an ounce of tincture of opium, administered. He had been bled *ad deliquium* in garrison, and was twice bled in hospital; about twenty-five ounces being procured from the last bleedings. A second dose of the calomel and opium was given, and soon after he fell asleep. Head-ach came on, and a muddy suffused state of the eye; but all the urgent symptoms of cholera disappeared. The second case was also relieved by venesection, and the use of calomel and opium in more moderate doses. In a third case, I tried the experiment of an emetic, with the warm bath, conceiving that the disorder had its origin in a defective action of the heart and lungs; or believing that the stimulus of artificial vomiting would enable these, and the adjoining viscera, to relieve themselves of the load of blood which impeded the free exercise of their functions: but in this I was disappointed; the symptoms, if not aggravated, certainly received no mitigation from the remedy; and the patient afterwards died, on the evening of the following day.

The symptoms which succeeded in the other two, as they belong to the stage of *febrile re-action*, which always occurred where the patient was not cut off early, will most properly be considered along with the description of that form of the disorder.

*Second form, or stage of oppression and exhaustion.*—When this stage of the disorder first occurs, it is not easy to determine: in some cases it may exist from the commencement, and certainly in by far the majority visited by the medical practitioner, is the one first presented to him. The cases received into my ward in this stage amount to ten; and about the half of that number had been affected with decided symptoms of the epidemic for eight hours previously to admission; one had been ill thirty-two hours, and still the symptoms of oppression and exhaustion continued, without any febrile re-action; another, twelve hours; a third, six; and two cases occurred where only two hours had elapsed from the first attack: yet, in all these instances, so various in their period of duration, the same, or nearly the same, symptoms were observed; and the relation of one may be considered as a history of the whole.

Nothing is more characteristic of this horrid disorder than the *expression* of the patient, as displayed in the altered state of his countenance and general aspect. This change never fails to strike an observer, and is said to be one of the earliest indications of the attack; but, as I have seldom seen a case before two hours had elapsed, I am not prepared to refer to it as contemporaneous with the first deviation from health, but must consider it only as occurring in the stage which I have here denominated second in order. It is impossible, I think, to mistake the *facies cholerica.* The countenance is in a slight degree livid, with a sort of leaden paleness intermixed; occasionally, the livor more marked in the lips and at the point of the nose; and a blackish or blue circle around the eyes, the ball itself appearing sunk in the orbit; and the muscles and cellular substance of the cheek wasted and fallen close over the bone; presenting altogether an aspect of ghastliness and death, which the pencil only can pourtray, no language being adequate to convey an idea of it. The patient moves from side to side, languid, yet solicitous to obtain relief from change of posture, which the little strength left to him can barely effect; and seems overpowered by some oppression of the internal organs, apparently of the heart and chylopoietic viscera; the eye, in general, remaining quite clear, and sometimes even lively to the last. The surface is cold and bedewed with a clammy moisture, and the thirst in all intense and unquenchable. The pulse, in general, is barely perceptible at the wrist, beating from 116 to 125, distinct and regular, but small and very feeble, or thready. In one case, it was quite gone; and in another not only perceptible, but possessed a tolerable degree of strength. Pain at the epigastrium and in the sides, though a frequent concomitant of this stage of cholera morbus, was by no means general in the cases admitted into the hospital: three only complained of a degree of pain in the region of the stomach or liver. In one of these it was very trifling, but increased upon pressure; in another, the sense of pain was deeply seated; but all the patients referred to some sort of uneasiness at the upper part of the abdomen and lower thorax, which they could not describe. It was not pain, but seemed to consist of anxiety and oppression, causing sometimes impeded respiration, and in almost every instance a moving from side to side in bed, as if change of posture would bring relief. One man complained he could not see, but he recovered his sight after blood-letting. In another, a slight degree of head-ach occurred; and this symptom may have been present in several, but, from its not being characteristic of the disorder, it had not been particularly recorded. Spasms of the hands and legs, or of the inferior and superior extremities, occurred in all but two; and one was attacked with severe general cramp. Occasionally the thighs became rigid; but the most common seat of spasm seemed to be the gastrocnemii muscles, and those of the fore-arm, causing clenching of the fists and a rigid extension of the feet. The vomiting and purging, which took place at the commencement, had generally abated by the time the patients were brought to the hospital; and in none of the cases did these symptoms prove severe. Occasionally a slight retching remained; and, where there was purging, the matter evacuated consisted of a watery fluid, tinged of a whitish colour, sometimes resembling thin oatmeal gruel or chicken soup, and at other times water, with some flocculi of rice swimming in it. The tongue, whereever it was noticed, was of a dirty white or clay colour, but moist.

On a general review, from recollection, of the cases admitted into the hospital, the most striking and characteristic features of the disorder consist of the extreme debility,—the alteration of the countenance,—the restlessness of the patient,—his feeble exhausted voice, and frequent calls for water to allay the excessive thirst which torments him. This last, probably, created more distress than any other symptom, or than all the others put together, as it could never be indulged, from a risk of exciting vomiting. The impression produced on the bystanders from all these circumstances, was that the vital organs were labouring in vain to rid themselves of some burden, and that, already nearly overpowered, the patient must soon fall a victim to the violence of his disorder. How strictly this was verified, may be seen from the results of the cases from which the above description is taken,—not one having recovered. In general, they survived from six to eight hours after admission into the hospital; from ten, to fifteen or twenty from the first attack, as far as could be ascertained by their own statements, and the inquiries made at the time. One survived thirty-six hours, another thirty; and a third case died in about nine hours from the occurrence of the first unequivocal symptom of the disorder. It almost always attacked them early in the morning, or during the night, and in some instances about bed-time.

The course of the disorder in these deplorable cases, after admission into the hospital, was generally marked by an increase of the weakness, languor, and restlessness; while the more urgent symptoms, as vomiting, purging, and cramps, were relieved, or altogether removed, by the opiates and other remedies employed. One symptom, the urgent thirst, remained to the last; and the poor patient, with a feeble, hardly articulate, voice, was incessantly calling out for water to relieve him. In some of the cases, the body became warmer, while the expression of the countenance remained as unfavourable as ever; and, although the patient himself declared he felt better, it was too evident to the by-standers that the disorder had not abated in the least. The features always retained their original ghastliness, and seemed to have settled in death long before the laboured breathing of the patient announced that his spirit was about to take its departure for ever. One man, in whom the general course of the symptoms and issue were similar to the others, complained of severe pain about the kidneys. Almost all retained their sense to the very last, though they were evidently not so acute as in health; and a striking indifference as to the event of their disorder appeared to prevail in the minds of every one. His present bodily suffering was, indeed, so great as to absorb every feeling which might have been excited for the result of the attack; and if any fears respecting this, arising out of the well-known fatality of the epidemic, existed at first, they certainly were forgotten before the malady reached that aggravated form which was presented to us.

With respect to the treatment of the disorder, little satisfactory can be said, though the trials of remedies were many and various, and the boldness and perseverance displayed in the use of them not to be exceeded.

On first seeing a patient whose appearance argued a certain degree of strength yet remaining in the circulation, our practice was to order blood-letting from the arm immediately. Two cases of this description occur, in one of which eighteen ounces of blood were procured, and about four or five in the other; but in both it flowed languidly, and was of an oily appearance, and darker colour than usual, congealing afterwards into a tar-like mass, without undergoing a separation into two parts by proper coagulation. No benefit was, of course, derived from the depletion, but the symptoms were not at the time aggravated by it. Topical bleeding, by means of leeches to the epigastrium, was tried in one or two cases, partly with a view of relieving the oppressed state of the circulation generally, (as where the blood would not flow from the arm,) and partly in the expectation of the local congestion being thereby diminished; but in no instance did it fulfil these intentions. Next to bleeding, greatest reliance was placed in opium, which was exhibited in all its forms. The tincture, given by the mouth in drachm doses, or combined with ether, was generally rejected; and I found a watery solution of five, and sometimes ten grains of opium, much more efficacious in allaying the irritability of the stomach, and removing the cramp. This quantity, dissolved, or rather suspended, in a few drachms of water, was ordered to be repeated every half-hour, if the cramps or purging continued. After taking one or two doses, the heat sometimes returned, and the pulse became stronger, and the peculiar narcotic influence of the medicine was clearly displayed; but the same issue took place as in the others. I certainly think that the opiates in several prolonged, though they could not ultimately sustain, life; and they are entitled to rank next to venesection in every stage of the disorder hitherto considered.

Clysters, composed of from three drachms, to half an ounce of laudanum, with a large quantity of warm water, were administered, and might have aided the effects of the opiates by the mouth; but this form of administering the medicine was not followed by the same manifest advantage as I had observed in the cases of natives formerly under my care. Such injections were repeatedly thrown up, in combination with the other remedies; and sometimes a few ounces of Madeira wine were mixed with the laudanum, to act as a general stimulus, and with a view of furnishing nourishment where the case was protracted, and the irritability of the stomach continued great. In one or two cases, where a degree of stimulus was judged proper, turpentine was added to the enema; but no good effect was observed from this, any more than from the other means employed. The primary indications which presented themselves in this form of the disorder, were to allay the spasms and vomiting where this symptom existed, and to remove the oppression. Where bleeding, opiates, and the other means now enumerated failed to accomplish these, we were compelled to have recourse to stimulants to support life, although, on the general principle, they might appear prejudicial. Those commonly used were brandy and water, alternated or combined with compound spirits of ammonia and ether. Sinapisms, too, with the same view, were applied to various parts of the body: sometimes they reddened the surface and gave pain, and at other times caused no change whatever.

Where calomel was given, it was always combined with opium, in the proportion of two of the latter to eight or ten grains of the former; and I think it sometimes did good.

All regular, *rational* practice failing, we were glad to adopt such empirical modes as were recommended by individuals of the profession, or found their way to public notice through anonymous writings in the newspapers. Among these we may reckon camphor, castor oil, and magnesia; all of which were tried, but certainly not so extensively as to furnish any grounds of judging respecting their remedial powers in the disorder. The first of these, it was reported to me, had been successfully used by a medical friend, who prescribed it in the dose of fifteen grains, repeated. In the case where I tried it, it was taken in the dose of seven grains and a half, combined with two of opium, every half hour, while the symptoms continued unabated. The patient took it nine times; became better; his voice was stronger: but these good effects were only temporary, for he, like all the others, died in the end.

Castor oil, it was said, had been given with great success to the Europeans in the grand army of 1817; and I naturally thought of it when other remedies failed. In one case, it certainly remained on the stomach when every thing else was rejected; but my experience of its efficacy being confined to this single instance, cannot form any grounds of induction, and offers, indeed, little to hope for, as the patient ultimately fell a victim to the disorder.

Magnesia, given in milk, was first announced in the papers as a certain specific for the disorder: to it, also, I betook myself, not with the expectation of its publisher, but merely that nothing might be left untried which was not repugnant to common sense. A drachm was given in four or five ounces of warm milk, and repeated with advantage. The patient appeared better for a short time, but no permanent good effects were derived from it, any more than from the others.

It were useless to state the remedies that suggested themselves as applicable to the condition of the patient, when contemplating him labouring under this dreadful disorder. Almost all which were available had been already tried by others, and it seemed unnecessary to repeat them. Two, however, occurred to me as peculiarly calculated to afford relief from the excessive oppression at the præcordia, which in many cases formed the only distressing symptom: these were galvanism and the inhalation of oxygen gas. The apparatus for either of these not being at hand, and demanding more manipulation than Indian means commonly admit of, I never could put them to the test of experiment; but, from reasoning on the subject, they certainly promise to be useful as stimulants of the heart and vital powers. On the same principle, the nitrous oxide gas might be successfully inhaled.

*Appearances on dissection of the body, in the first and second stage of the disease.*—Only three dissections occur of those who died in the stages now described. They do not furnish any very conclusive evidence respecting the nature of the disorder, but, as differing from the state of parts in the protracted form of the epidemic, they may be stated here. On the inner surface of the stomach were observed small red spots, but no decided marks of inflammation. In the duodenum and jejunum nothing unusual, excepting perhaps a slight blush; but, in the ilium, such an increased redness and enlargement of vessels were noticed, as must be set down to the effects of incipient inflammatory action. In one of the cases, the gut was covered internally with a whitish lymph, or puriform humour, resembling what is more frequently observed in the latter stage of the disease. The gall-bladder, in two cases, was distended; and, in the other, only half filled with a thin dark-green bile. The liver and other abdominal viscera quite healthy. In the thorax, the right auricle was enormously distended with a black, coagulated blood, and the veins about the heart were of a larger size than usual. The left side of the heart quite collapsed, and the auricle rather diminutive. These last circumstances more particularly attracted our attention, as they appeared connected with the extraordinary anxiety and oppression, which formed so characteristic a symptom of the disorder.

*3d, or stage of febrile re-action.*—I denominate this protracted form of the epidemic the stage of febrile re-action, as it resembles very closely the common Bengal remittent fever, and as the symptoms ushering it in are indicative of considerable general irritation, which may not unreasonably be attributed to an effort of the system to free itself of the previous oppression and disorder in its several parts.

This stage generally occurred from thirty to fifty hours, or about two days, after the first attack. The altered condition of the discharge from the bowels was considered as pointing out the accession; and, where great torpor of the intestines was present, the increased temperature of the surface, and more natural pulse plainly denoted that a change had taken place in the general functions. Still, great weakness prevailed, and in almost all cases an excessive listlessness, which looked at times like insensibility; but any delirium at this period was very transient, and it occurred in one case only. The looks became more natural, the surface more dry and hot; and the pulse, though still feeble, was fuller, and beat about 96 in the minute. In one or two cases it was very full and strong, which may have been occasioned, in part, by the opiates taken in the preceding stage, the secondary effects of which were obvious for a day or two. Tongue moist at the edges, of a deep, dark fur, and dry in the middle. Two or three days elapsed, in some instances, before medicines could be got to produce any effect on the bowels; and in others the stools were copious from the first, and always of a dark green, almost black, colour, and liquid consistence like vitiated bile, and resembling in every respect the discharge from the bowels in the common form of the Bengal remittent. In two or three instances vomiting came on in the course of this stage, but it never proved lasting nor severe. The matter vomited resembled that discharged by stool, and was evidently composed of vitiated bile. Where recovery ultimately took place, the pulse became more natural, and, indeed, never was so weak and quick as in the fatal cases. The strength did not decrease; and, the functions gradually returning, the patients were discharged in ten or twelve days from the date of admission into the hospital. In the bad cases, the progress of the disorder was marked by an increase of the frequency and feebleness of the pulse, and a progressive debility; but the event occurred at very different periods. One man survived only two days after the accession of the febrile stage; another, four; and a third lived to the seventh day, or six inclusive. In this last case, hiccup and coffee-coloured discharge and vomiting came on two days before death; but, what was rather remarkable, the day immediately preceding this termination the stools became of a natural appearance, and the other symptoms seemed more favourable.

The restlessness was sometimes very great, and the patient either lay quite indifferent to every object around him, or tossed from side to side in a languid manner, presenting to the bystanders an object of great distress, though less powerfully affecting than in the former stage, when the suffering from the cramp was so very acute.

In one case, where the symptoms were far from severe, a sudden aggravation took place without any visible cause: the pulse, from being pretty good, became quick and feeble, the body cold and clammy, and dissolution followed on the evening of the same day. One patient complained of pain in the left side, and in two other cases there was slight head-ach; but, in general, the sensibility appeared blunted, and pain formed no part of the symptoms.

The treatment adopted in this stage had in view the removal of the vitiated bilious discharge, and a change to a healthy condition of the disordered functions causing it. I regret to add, in no case which had not been vigorously combated at the early periods of the attack, was this practice successful. With the exception of the instances alluded to under the head of the second stage, all the patients equally fell victims, more slowly indeed, but not less surely, to the violence of the malady. Purging, and mercurial inunction, were resorted to in most cases; or calomel was regularly administered every two hours, sometimes with opium, but in general without it, or in larger doses (℈j. or gr. xv.) three times a-day. In one case it was given in the dose of six grains every hour, at the same time that mercurial ointment was freely rubbed in, over every part of the surface, and a blistered surface over the stomach dressed with it. In one of the fortunate cases, where the patient had been freely bled during the first stage, and taken calomel in half-drachm doses, half an ounce of the ointment was rubbed in twice a-day, and the submuriate continued in the dose of ten grains. On the second day the mouth became affected, when the remedies were intermitted; and he took afterwards a light infusion of cherayter. Once, in the progress to complete recovery, a determination of blood to the head occurred, which was removed by local bleeding.

The purgatives employed were rhubarb and magnesia in combination, jalap and calomel, scammony and calomel, gamboge and calomel, and infusion of senna with salts. In two of the cases, very large doses of jalap and calomel were given, without any operation on the bowels; and glysters of senna and salts and aloes were thrown up the gut. One of turpentine combined with castor-oil was recommended to me as an excellent and efficacious cathartic in those obstinate cases; but, not having an opportunity of trying it in a proper subject, I cannot myself speak as to its merits. At the same time that purging and the mercurial practice were freely employed, it became necessary to consider the debilitated state of our patient, and to endeavour to supply nourishment in the lightest and most digestable form. Soup, and wine and water were frequently given, the latter in a regular manner; and, when the stomach was irritable, mutton-broth was injected by the anus; but it appeared, in the fatal cases, as if no absorption took place from the stomach or any part of the intestinal canal, for I did not find an instance in which yellowness of the eyes or skin occurred, notwithstanding the great abundance of the bilious discharge, and its presence at all times in the stomach and intestines: and in this respect, perhaps, the disorder may differ materially from the state of fever which it otherwise so much resembles. When the strength was much reduced, and the patient apparently sinking, brandy and water were given, combined occasionally with red pepper (Cayenne), and sinapisms were bound to the feet. Ether, in one case, was administered; and turpentine glysters were also used, without any change in the symptoms. In a solitary instance I made trial of the cubebs pepper, to which I was induced by the appearance of the gut (covered with a thick mucus-like gonorrhœal matter,) found on dissection of a former case. It was given in the dose of fifteen grains every two hours, but vomiting following, it was immediately discontinued.

*Appearances on dissection of the body in the third stage.*—The body was always opened early after death, that no change might occur from putrefaction. Five cases were examined, where the patients had died in the last stage of the disorder. In one of them only was the head opened. The membranes of the brain were found of a natural colour, with the exception of a dimness of the pia mater at particular points, which, on a more minute examination, seemed to proceed from a greater proportion of serum on the blood-vessels, and a deficiency of red globules. No signs of congestion or previously-increased action of the vessels of the brain were visible; and there was no greater quantity than usual of serum effused.

In the abdomen, the omentum in one case was found drawn up to the stomach, and of a redder colour than natural. The stomach itself, in every instance, slightly reddened externally, and generally contracted; in one case, so much so as to resemble the contractions which are occasionally met with on the intestinal canal where no organic disease has existed. On cutting into the bag, the whole inner surface was found of a reddish colour, in some cases florid, but in others approaching to that of rust of iron; not stellated, as occurs in cases of cholera where the patient had died soon after the attack. The colour was always most intense towards the pylorus, and extended from thence into the duodenum. It appeared that this inflammatory redness was confined entirely to the mucous coat of the stomach, as it could be removed by scraping the surface with the scalpel, and the muscular coat so exposed presented no unusual colour. There was, in general, a dirty watery fluid contained in the stomach, with small greenish or blackish flakes swimming in it, which adhered to the mucus lining its inner surface. The small intestines externally did not differ much from their usual appearance, excepting in one case, where they were of a purple colour, and here and there of a florid red; internally, the same redness was observed as in the stomach; and when the mucus was cleared away, and the tube cut out and held between the eye and the light, the enlarged and numerous blood-vessels looked like a dense net-work, and an approach to ulceration was perceived at several points in one instance. The gut contained a yellowish coloured, thin, bilious excrement, which, like the black flaky substance in the stomach, adhered closely to the mucus lining its inner surface. Sometimes the mucous secretion of the intestines seemed changed into a puriform humour resembling the matter of gonorrhœa. All these appearances, with one exception, were confined to the small guts, the colon not participating in the diseased structure. In one instance, the caput cœcum was unusually large, and lay over the bladder at the lower part of the abdomen: its inner surface was reddened, too, like that of the small guts; and in another case, complicated with chronic dysentery, the great gut, in its whole length, presented a diseased secretion and structure. At the lower part of the ilium, the mucus was of a red colour, like thin coagulated blood; and this appearance continued all the way to the anus: the coats of the gut were also thickened, and otherwise altered in structure, as is usually observed in dysenteric cases of long standing. The liver, in all the cases, appeared to contain more blood than usual; and in one was harder, and showed on its surface several puckered-looking points, resembling old cicatrices; but it did not, in any instance, exceed the common size of that organ. The gall-bladder, in general, was distended with a thin green bile; in one it was only half filled; in another, the bile was more viscid and ropy; and in a third, it was enlarged to twice the common size, and completely filled with a transparent colourless liquid, like water having dissolved in it a small quantity of pure mucilage. The vessels on the internal surface of the bag in this case seemed more enlarged than usual, and their contents were of a darker colour. The cystic duct was also completely obstructed, and much thickened. The spleen, in one case, was rather smaller than usual, but in the others quite healthy and natural in every respect; as were all the other abdominal viscera.

The cause of cholera morbus, in the cases from which the preceding history has been taken, could not be ascertained. The subjects were chiefly men in the prime of life, soldiers and sailors lately arrived from Europe. They had been exposed to the sun, walking the streets of this city, or on-board ship; and many of them, too, had been drinking very freely of bazar liquor, which is a spirit in general possessed of the most noxious qualities. As far as observation goes, the prevalence of the disorder appeared in some degree connected with a certain state of the atmosphere, and cases were most frequent upon any change of weather. Dense, detached clouds were common precursors of cholera.

